# Education in psychiatry: the art of getting published

**DOI:** 10.1192/bji.2021.27

**Published:** 2022-05

**Authors:** David H. Skuse

**Affiliations:** Professor of Behavioural and Brain Sciences, Division of Population, Policy and Practice, UCL Great Ormond Street Institute of Child Health, London, UK. Email: d.skuse@ucl.ac.uk

**Keywords:** Composition, publication, citations, journal, academic

## Abstract

Getting an article published in a scientific journal requires skills that are rarely taught, but are almost invariably learned by (bitter) experience. Yet, there are generally applicable guidelines that facilitate the process. This article summarises them.

## Giving clinicians a voice

There is an ‘art’ to getting your article published in a scientific journal. Many aspiring authors do not understand, probably because they have never been told, that it is not sufficient just to have a good idea. There are some general ground rules, and there are certain inflexible limitations, which differ from journal to journal. But beyond those, there is the need to consider basic questions, such as why do I want to publish? What is my message? Am I doing this simply to get promotion or retain my post? Sadly, in these days of predatory journals that advertise the opportunity to publish almost anything (at a price), we are at risk of demeaning the noble art of writing scientific articles in pursuit of quantity rather than quality. No country is entirely free from the menace of universities that link your career prospects to your *N* of publications or to the citation index of the journals in which you publish. There is no simple escape route from this dilemma. Working collaboratively, in an area of research that attracts lots of attention, will benefit those who are in large teams. They are likely to produce more – and more highly cited – publications. We at the *BJPsych International* have a mission to give a voice to clinicians and clinician-scientists who do not have access to those facilities, but who nevertheless are doing work that benefits patients, directly or indirectly, with novel approaches that others could emulate. Although we do not publish original research, our mission is to educate; it could be construed as hypothesis generation, work that could be rigorously evaluated by scientific enquiry by others with the resources to do so.

## Where to start

### The abstract

The first question to ask yourself when composing an article for our journal, or any other reputable publication, is ‘What is my message?’. That might sound facile, but it is not at all easy to answer for most aspiring authors. Your message should be simple. Remember, however good your article is, your reader is likely to remember just one thing from reading it. You can quite easily identify whether or not you have a simple message by writing an abstract. Good abstracts are about as difficult to compose as your entire article. My recommendation to any aspiring author is to compose your abstract before you write the paper. Show the abstract to your colleagues and ask them, ‘What do you think this article is about? What is the take-home message?’ If they are unable to give you an answer straight away, you will have to redraft it. When writing your abstract, remember you need to have identified the journal to which you will submit, and you should have looked at the formatting of the abstract required by that journal. You should be aware of its length. Many journals advise an upper word limit for the abstract. Can you get your message across in less than 200 words?

### Choosing the journal and checking its instructions for authors

We have discussed the length of the abstract and the fact that it should encapsulate the essence of your article. Another general point about publication is the choice of journal for your message. How do you decide? I have already emphasised the absurdity of judging the worth of an article on the citation index of the journal in which it is published. That index will correlate positively with the difficulty of getting your article accepted, but it is no guarantee that anyone will want to cite it. In the days of search engines such as PubMed and Google Scholar, if your article is indexed, it will be accessible to anyone. It does not matter if no library in the world stocks the journal in paper form. If what you wrote is worth reading, it will be cited. I know authors who are now Fellows of the Royal Society, who have barely published a single article in a high-impact journal. That said, if you work in an obscure area of science your work is less likely to be searched for than if you are in a popular area. As a general rule, highly cited articles are often original and authoritative reviews, or the description of a new methodology for measuring something (in our field, it is often a questionnaire or an interview).

Avoid piecemeal publication: your article should deliver just one message. You may find there is a good reason to divide up your findings from a substantial piece of research because they will fit more appropriately into a range of specialist publications. Which leads on to another point that is often ignored by prospective authors: read the instructions for authors that are published by the journal on their website, and make sure your article fits with the aims and objectives of the journal! Look for articles that are similar – if no article on that subject has been published in that journal it is unlikely to accept your article either, so you should look elsewhere.

It is incredibly irritating, as an editor, to receive an article that is formatted in a way that bears no relationship to your journal's format. The article is the wrong length (often two or three times too long), the referencing is wrong, perhaps even the content is inappropriate. The editor will assume it is an article that was rejected by a previous journal and has simply been passed on without any further consideration. This is insulting to the journal and guarantees instant rejection.

### Writing: style and content

Take an objective look at your writing style. Avoid excessively complex and repetitive language. Try to avoid overusing certain words: for example, there is a temptation to stick ‘However’ or ‘Nevertheless’ at the beginning of sentences. So do a count of how many of these words are in your article. It is easy nowadays with a program such as Word. Cut them out. Use short sentences. Avoid excessive verbiage. Journals are short on space; they have page budgets. Keep it brief. When it comes to citing literature in your article, make sure you have actually read the references – and make sure they are accurately quoted. I have often looked up what appears to be a promising reference, only to find the article cited says no such thing. My guess is this is because the original article was cited by another paper, and a lazy author took the citation straight out of context without checking it. Don't copy what someone else said a reference claimed without reading the original yourself and checking. If you cannot find the original reference, don't use it at all. Make sure your references are up to date. This is a particular bugbear of mine and bedevils work in our area of medicine. Although psychiatry sadly evolves at a snail's pace compared with fields such as immunology or cancer, it is usually pointless discussing findings in relation to publications from 20 years ago if the field has moved on and definitions have changed.

If the journal in which you are publishing accepts tables and figures, know the difference. A typical table provides a summary of data in rows and columns. Figures, on the other hand, are illustrations, for example flow charts or histograms. Composing either is challenging. Show your colleagues. Are they clear? Could you understand them without reference to the text? Don't prepare coloured figures if the journal only publishes in black and white.

Finally, the discussion section always requires a certain format, irrespective of the journal. New information is not appropriate for the discussion. Don't put results into the discussion unless they have already been presented. The point of a discussion is to review the findings and draw out the most important aspects of your text. It should be structured. It should set your findings in the context of existing literature. Limitations are an important aspect of the discussion. How could your study be improved? What have you learned from your investigation? What are the implications for generalisation? What are the implications for the future?

## The Times They Are A-Changin’

Changes in publication practice are coming thick and fast. There is a trend for prepublication in an online format, inviting criticism from your peers, prior to submission to the journal of your choice. One such preprint server is the bioRxiv (pronounced ‘bio-archive’) (www.biorxiv.org). A complementary system, which works well for many scientific investigations, is the registered report. This is a publishing format used by an increasing number of journals, in which the study design is published ahead of data collection and is subject to its own peer review. Not all articles are suitable, of course, especially commentaries and exploratory studies. But, as the now 80-year-old Bob Dylan said, ‘The Times They Are A-Changin’.

